# Enhanced Degradation of Waste Activated Sludge in Microbial Electrolysis Cell by Ultrasonic Treatment

**DOI:** 10.3389/fmicb.2019.00128

**Published:** 2019-02-05

**Authors:** Kai Hu, Wei Chen, Shuo-qiu Jia, Wei Wang, Feng Han

**Affiliations:** ^1^Ministry of Education Key Laboratory of Integrated Regulation and Resource Development on Shallow Lakes, Hohai University, Nanjing, China; ^2^College of Environment, Hohai University, Nanjing, China; ^3^Hydrology & Water Resources Bureau of Henan Province, Zhengzhou, China

**Keywords:** waste activated sludge, microbial electrolysis cell, ultrasonic pretreatment, hydrogen, organics degradation, microbial communities, organics fractionation

## Abstract

This study investigates the feasibility of ultrasonic pretreatment for improving treatment efficiency of waste activated sludge (WAS) in microbial electrolysis cell (MEC). Results showed that at applied voltage of 0.5 V, biogas production and cathodic hydrogen recovery enhanced 3.68-fold and 2.56-fold, respectively. Due to the transformation of soluble COD accelerated by the pretreatment, the removal rates of suspended solids and volatile suspended solids were significantly enhanced by 1.38-fold and 1.48-fold, respectively. Various kinds of organics, including VFAs (volatile fatty acids), proteins and carbohydrates, could be utilized in sequence. The primary biodegradable substance in MEC was hydrophilic fraction from sludge organics and the pretreatment effectively resulted in an elevated concentration of this fraction. The 16S rRNA pyrosequencing analysis demonstrated multiple syntrophic interactions between fermentative bacteria, exoelectrogenes, and methanogenic archaea in MEC for WAS.

## Introduction

Globally there is a strong consideration toward WAS, which is massively generated by commonly used biological wastewater treatment process, as a source of renewable energy, i.e., named biomass. This conception was emphasized by the reality of shortage of energy and concerns over greenhouse effect (Hasan and Rahman, 2017; Abdulrahman and Huisingh, 2018; Uzoejinwa et al., 2018).

Anaerobic digestion was an anaerobic approach to stabilize sludge, while simultaneously produces biogas to offset part of the consumption. The main constituent of biogas produced by AD is methane (accounted for 55–65%). The other constituents include 30–40% of CO_2_, fractions of water vapor, traces of H_2_S and H_2_ (Appels et al., 2008). Methane is regarded as an important energy source. However, in terms of energy content, hydrogen (141.8 MJ/kg) has a 2.6 times over methane (55.5 MJ/kg), which is a favorable energy carrier. Moreover, “fermentation barrier” is an issue during AD process that causes fermentation dead-end products accumulation (e.g., acetic acid) (Zhu et al., 2014). Typically, a secondary aerobic process was required to decompose these residual organic matters; In this case, energy containing in these organic matters was wasted. MEC, a new anaerobic biotechnology, is reported to be an alternative way to solve this problem and simultaneously recover hydrogen. Various substrates were used in MEC to produce hydrogen, including VFAs, glucose, glycerol, cellulose, protein, and etc. (Kadier et al., 2014). In recent decades, more attentions are paid to real wastes, e.g., domestic wastewater (Ditzig et al., 2007), swine wastewater (Wagner et al., 2009) and sewage sludge (Lu et al., 2012; Guo et al., 2013; Sun R. et al., 2014). These studies indicated the feasibility of waste treatment via MEC technology. Nevertheless, most studies tested sludge fermentation liquid as substrates for the MEC feasibility. These researches separate the fermentation process from microbial electrolysis process. The WAS, with its abundance in nutrients and carbons, is a quite complex waste. Interestingly, a mixed culture was required for achieving better performance rather than pure culture (Liu W.Z. et al., 2016). In this opinion, microbial electrolysis process could combine with fermentation process for sludge treatment. During this process, it is very important to reveal how the multiple groups of microorganisms work together to transform complex substrate to energy products, such as hydrogen or methane.

The main organic compounds in WAS were EPS and IS, both of which included lipid, polysaccharide, protein, and nucleic acid (Appels et al., 2008). These compounds are not readily to hydrolyze. Accordingly, pretreatment method was recommended to disintegrate sludge matrix and release these compounds to the solution. One of the methods gaining increasing interests is the use of ultrasound due to its advantages such as shorter treatment time, no chemical addition, and no generation of by-products (Li et al., 2018). Ultrasonic sludge pretreatment is based on the influence of cavitation derived from collapse of microbubbles, which results in deagglomeration of flocs, microorganism cells disruption (cells lysis), sonochemical effects, and rheological changes (Skórkowski et al., 2018). Most laboratory and industrial scale studies reported enhanced performance by this method before AD or fermentation process (Zhen et al., 2017). The characterization of community structure and community shifts when fed with U-SS is not well understood. However, it starts to attract interest of scientists and engineers.

In this study, ultrasonic pretreatment was chosen to disintegrate WAS and the influence of U-SS on MEC performance was investigated. To achieve this goal, raw sludge was compared with U-SS as substrates in a single-chamber MEC. Besides, the hypothesis that sludge utilization can be directly achieved for hydrogen production in MEC instead of sludge fermentation liquid was explored in the present study. From this aspect, variations of both soluble organics and EBOM in influent and effluent from MEC were examined using hydrophilic-hydrophobic fractionation and EEM fluorescence spectroscopy. The microbial community structure of anode biofilm was analyzed using high-throughput 16S rRNA pyrosequencing in order to relate reactor performance with microbial function, with emphasize on the microbial behavior and interactions.

## Materials and Methods

### Raw WAS and U-SS

Raw WAS used in this study was collected from a secondary sedimentation tank in a municipal wastewater treatment plant located in Nanjing, China. The ultrasonic pretreatment was conducted as follows: firstly, pH of WAS was adjusted to around 10 using 5 M NaOH solution. This step was on the basis of the fact that alkaline condition promotes the hydrolysis of proteins and fats and could be combined with the ultrasound method to enhance the sludge solubilization (Carrere et al., 2016). Then the WAS was treated for 5 min at an ultrasound energy density of 3 W/mL by a laboratory ultrasonic cell disrupter (JY98-IIIN, Ningbo Scientz Biotechnology Co., Ltd., China). Before being fed into MEC, pH of U-SS was adjusted to approximately 7 using HCl solution. The characteristics of raw WAS and U-SS were presented in Table 1.

**Table 1:** The characteristics of raw WAS and U-SS.

	SS (g/L)	VSS (g/L)	TCOD (mg/L)	SCOD (mg/L)	Dissolved protein (mg/L)	Dissolved carbohydrate(mg/L)	pH	Conductivity (mS/cm)
WAS	7.61	4.86	8954	46	2	2	7.3	1.1
U-SS	6.58	3.07	8638	1039	175	82	7.2	5.3


*SS, suspended solid; VSS, volatile suspended solid; TCOD, total chemical oxygen demand; SCOD, soluble chemical oxygen demand.*

### Reactors Configuration

At first, a two-chamber MFC was employed for anode biofilm enrichment. The MFC was H-shaped with two identical cylindrical chambers (7 cm diameter × 7 cm length, effective volume of 269 mL) separated by a proton exchange membrane (N117CS, Dupont Co., Ltd., United States). A carbon fiber brush (5 cm diameter × 5 cm length, fiber type: T700SC-24K, Toray Co., Ltd., Japan) was introduced as the anode of MFC and in the case of cathode, a carbon cloth (WOS1002, CeTech Co., Ltd., Taiwan), coated with Pt catalyst (0.5 mg/cm^2^) was adopted. As for the MEC, the reactor was a rectangular shape (7 cm × 6 cm × 7 cm, effective volume of 294 mL). The MEC anode was replaced by the anode of MFC after enrichment and the cathode of MEC was exactly the same as that of MFC. Therefore, a single-chamber MEC was constructed.

### Inoculation and Operation

The MFC was initially inoculated with WAS. Then, a 50 mM nutrient phosphate buffer solution (1.5 g/L of CH_3_COONa, 2.41 g/L of KH_2_PO_4_, 7.35 g/L of K_2_HPO_4_⋅3H_2_O, 0.31 g/L of NH_4_Cl, 0.13 g/L of KCl, 12.5 mL/L of trace nutrient medium, and 5 mL/L of vitamin solution) was mixed with WAS at the volume ratio of 2:1. The catholyte of MFC was a 50 mM phosphate buffer solution. A resistor (1 kΩ) was connected with the MFC, and the voltage across the resistor was measured through a multimeter (DT-118, Shenzhen Everbest Machinery Industry Co., Ltd., China). Provided that the voltage reached a constant value, the MFC was refilled with new feed of mixed solution of WAS and nutrient phosphate buffer solution. When the maximum voltage was reproducible for at least three batch operation cycles, the biofilm on the anode was acclimated and then was transferred to replace the MEC anode.

The MEC was fed with raw WAS or U-SS in a 50 mM phosphate buffer solution (the same composition as MFC, except for the absence of CH_3_COONa). A fixed voltage (0.5 V) was applied to the MEC using a direct current power source (LP2002D, Shenzhen Lodestar Precision Tools Co., Ltd., China). A resistor (10 Ω) was connected with the MEC, and the voltage across the resistor was measured. A batch cycle ended when the voltage decreased below 10 mV, then the MEC was refilled. The produced biogas was collected by a gas bag (200 mL, E-Switch, Shanghai Shenyuan Scientific Instrument Co., Ltd., China), and the biogas volume was measured using a glass syringe. All tests were conducted at room temperature (22 ± 3°C).

### Analyses and Calculations

The influent and effluent of each batch cycle from MEC were withdrawn. TCOD, SS, VSS, SCOD and NH_3_-N were determined according to standard methods (APHA, 1998). The soluble protein, soluble carbohydrate and amino acid were examined using coomassie brilliant blue method, anthrone-sulfuric acid colorimetric method and ninhydrin hydrate colorimetric method, respectively, as described (Hu et al., 2018). The DOC content was determined by a total organic carbon analyzer (multi N/C 2100, Analytik Jena Co., Ltd., Germany).

The sludge sample was centrifuged for 10 min at 5000 rpm, then the sludge EBOM was extracted as described by Jiang et al. (2010). The EBOM solution and sludge supernatant were filtrated (0.45 μm) and acidified to pH = 2 using HCl solution. The organic matters in sludge were fractionated into five types: HPI, HPO-A, HPO-N, TPI-A and TPI-N, adopting XAD-4/DAX-8 resins (Supelco Co., Ltd., United States) as described by Xue et al. (2008).

The electrochemical performance of MEC was evaluated in terms of volumetrical current density (I_V_, A/m^3^), coulombic efficiency (*C*_E_, %), cathodic hydrogen recovery (*r*_cat_, %), and electricity efficiency (*η*_E_, %). The gas production was determined through production rate [Q, m^3^/(m^3^⋅d)]. The calculation methods for the above indicators were referred to literature (Logan et al., 2008).

### High-Throughput 16S rRNA Pyrosequencing

The microbial communities of anode biofilm and raw WAS were examined by high-throughput 16S rRNA pyrosequencing. The biofilm sample was obtained by cutting carbon fiber with a sterilized scissor when the current density reached maximum values. The WAS sample was centrifuged for 1 min at 1000 rpm and the supernatant was drained. All samples were stored below -20°C before analysis. The tests of DNA extraction, PCR amplification and pyrosequencing were conducted by Sangon Biotech Co., Ltd. (Shanghai, China), and the detailed information was available in the literature (Wang et al., 2017).

## Results and Discussion

### Performances of the MECs

**FIGURE 1:**
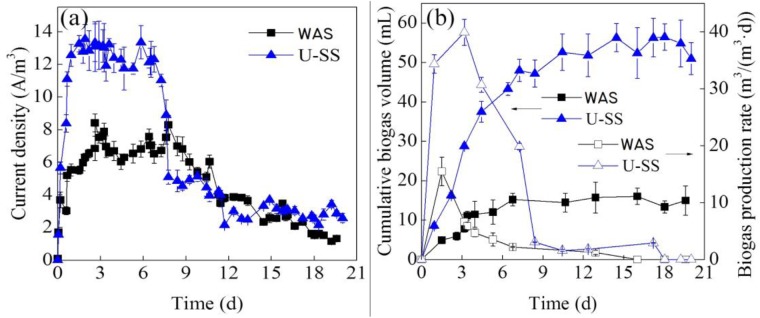
Variations of current density **(a)**, cumulative biogas volume **(b)**, and biogas production rate **(b)** in the MECs treating WAS and U-SS. (Error bars ± standard deviation were based on three MECs operated in parallel).

The performance of the U-SS MEC was superior to that of feeding WAS (Figure 1). With 0.5 V applied voltage, during a typical batch operation cycle, the current density increased dramatically at first. The maximum current density in the U-SS MEC (13.4 A/m^3^) was about 1.8 times of that in the WAS MEC (7.9 A/m^3^). Then the current density declined with time. At day 4, the current density in the WAS MEC decreased significantly; whereas in the U-SS MEC, the decrement delayed until day 9. Ultrasonic pretreatment resulted in solubilization of various organics from sludge matrix, hence accelerating the microbial electrohydrogenesis. After 11 days, the current densities in both MECs were averaged at 3.2 A/m^3^.

As can be seen in Figure 1a,b, the changes of current density were in accordance with the variations of biogas production rate. This result has been proved by the equation (1) (Logan et al., 2008), where Q represented volumetric hydrogen production rate, I_v_ represented current density, r_cat_ represented cathodic hydrogen recovery, F represented Faraday’s constant, and c_g_ (mol/L) was the molar density of gas at a standard temperature (298.15 K) and standard pressure (1 bar).

(1) $$Q=\frac{43.2I_{v}r_{\mathrm{cat}}}{{\mathrm{Fc}}_{g}(T)}$$

The MEC performance was listed in Table 2. The achieved TCOD removal rate was much lower than other studies (Liu W.Z. et al., 2016), which was above 60% adopting integrated system of microbial electrolysis and anaerobic fermentation fed with U-SS supernatant. This result implied the fact that the hydrolysis rate was the limiting factor in MEC in terms of COD reduction. Overall, the *C*_E_ values of both cells were not very high (33.7% for WAS MEC and 36.8% for U-SS MEC), and were comparable to the reported values of 28% ± 10% to 34% ± 4% (Lu et al., 2012). As a result of the suspended growth of microorganisms in WAS, a part of sludge organics was fermented and degraded regardless of applied voltage, which did not generate current. However, the ultrasonic pretreatment did not improve the *C*_E_ value.

**Table 2:** Performances of the MECs.

Substrates	SS removal (%)	VSS removal (%)	TCOD removal (%)	*C*_E_ (%)	*r*_cat_ (%)	*η*_E_ (%)	Q (10^-3^ m^3^/(m^3^⋅d))
WAS	25.5 ± 4.1	30.7 ± 3.7	22.0 ± 2.1	33.7 ± 10	5.0 ± 1.5	8.5 ± 2.8	2.5 ± 0.8
U-SS	35.3 ± 2.5	45.3 ± 3.6	29.7 ± 1.9	36.8 ± 6	12.8 ± 2.1	21.7 ± 3.8	9.2 ± 1.7


The *r*_cat_ value in the U-SS MEC was 2.56 times higher than that in the WAS MEC, but remained at a low level. The reasons could be ascribed as: (1) the consumption of H_2_ by H_2_-oxidizing methanogens; (2) the leakage problem; and (3) catalyst activity. Moreover, the complexity of substrate may lead to various reactions, which could influence the *r*_cat_ value (Cusick and Logan, 2012).

### Variations of Dissolved Organics

**FIGURE 2:**
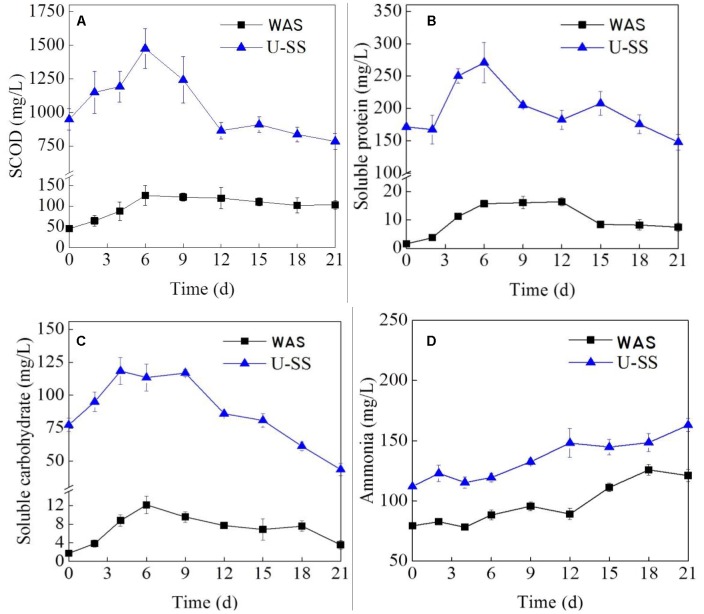
Variations of SCOD **(A)**, soluble protein **(B)**, soluble carbohydrate **(C)**, and ammonia **(D)** in MECs (Error bars ± standard deviation were based on three MECs operated in parallel).

During microbial electrohydrogenesis process, the SCOD variation is a function of solubilization and degradation of sludge matrix. Figure 2 presents the dissolved matter changes in the MEC.

At the initial stage, SCOD increased with time, implying a higher hydrolysis rate over degradation rate (Figure 2A). Noteworthy, the SCOD of U-SS was much higher than that of WAS and increased more rapidly, which was owing to the ultrasonic pretreatment. At day 9 (for U-SS MEC) and day 6 (for WAS MEC), the SCOD reached peak values and after that gradually decreased. This result was in consistent with the finding by Lu et al. (2012), which stated that from day 0.6 to day 8.8, the SCOD value almost kept unchanged in the WAS MEC and a rapid decrement of acetic acid was the main reason causing the SCOD drop. The SCOD changes were in agreement with the current density changes, demonstrating the relationship between electrochemical characteristics and organics degradation.

As for the soluble protein, the changes were identical to the SCOD changes (Figure 2B). The research conducted by Lu et al. (2010) confirmed the biodegradation availability of protein in the MEC, although obtained with a notable decreasing electrochemical parameters. In this study, in the latter stage of the operation, the protein concentration gradually declined, indicating the degradation rate grow at a higher speed than the hydrolysis rate. At this time, the readily-biodegradable organics concentration was low and protein became a major substrate.

The concentration of soluble carbohydrate rose at first and then declined (Figure 2C). A few part of exoelectrogens was found to directly utilize carbohydrate, such as *Rhodoferax ferrireducens, Klebsiella pneumonia*, and *Aeromonas hydrophila*, etc (Logan, 2009). Most exoelectrogens utilized carbohydrate on the condition that this material was subject to hydrolysis or fermentation. Therefore, at the first few days, the hydrolysis rate of carbohydrate was high, as can be seen in Figure 2C that showed an accumulation of carbohydrate. From day 9 for the both cells, due to the low concentration of VFAs, the microorganism turned to metabolize carbohydrates and proteins, resulted in an increment in the degradation rate. The above-mentioned results clearly presented how complex organics in sludge were converted to electrons in MECs, suggesting a cascaded utilization with time. This phenomenon was observed by many studies (Lu et al., 2012; Liu et al., 2012; Liu W.Z. et al., 2016; Wang et al., 2014).

A previous study showed the NH_3_-N reduction ranged from 174 to 186 mg/L in MEC fed with sludge fermentation liquid (Wang et al., 2014). If the MEC was dosed with complex substrate, for example WAS or U-SS in this study, the recalcitrant nature of complex waste caused an ascending NH_3_-N concentration (below 200 mg/L for both cells, seen Figure 2D) during the operation. The released NH_3_-N in the solution could not inhibit the microorganism growth.

### Transformations of Organic Fractions

**FIGURE 3:**
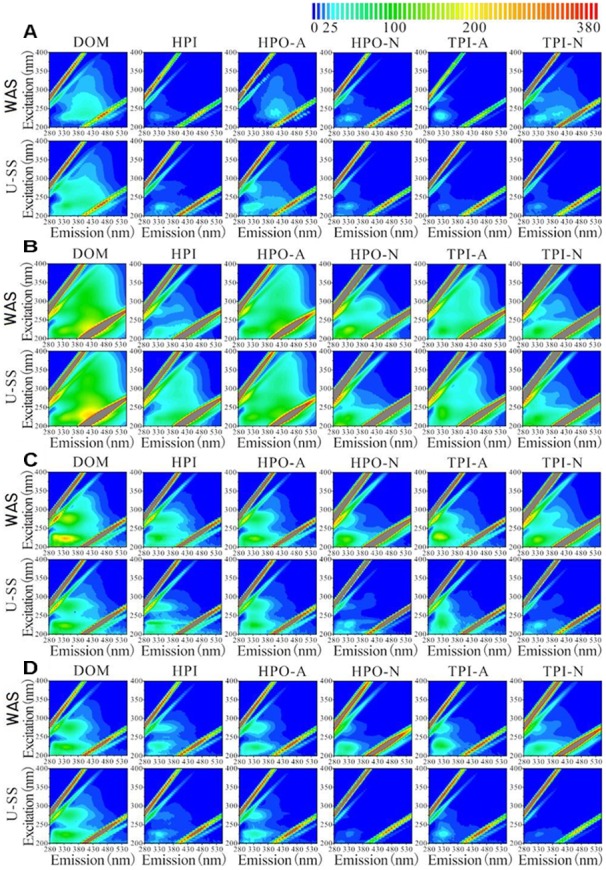
Excitation-emission matrix fluorescence spectra of DOM, HPI, HPO-A, HPO-N, TPI-A, TPI-N obtained from influent supernatant **(A)**, effluent supernatant **(B)**, influent EBOM **(C)**, and effluent EBOM **(D)** in MECs.

As indicated in Figure 3, the aromatic proteins and soluble microbial by-product-like materials were the principal components in influents and effluents. The influent (WAS or U-SS) EBOM of MEC contained few fulvic acid-like materials, while these materials were not detected in the effluent EBOM. Considering that all the effluent supernatant showed high fluorescent intensity in the region referring to the fulvic acid-like substances, these substances may be derived from the solubilization and metabolism of IS in sludge, not from EBOM. In the influent supernatant, U-SS exhibited higher fluorescent intensities in the regions referring to aromatic proteins and soluble microbial by-product-like materials, which demonstrated the effectiveness of ultrasonic pretreatment to break the sludge matrix and increase the soluble organics.

A weak fluorescent intensity of HPI fraction was found in the WAS supernatant. In the effluent of WAS MEC, the supernatant mainly consisted of HPO-N and TPI-N fractions, which were not readily-biodegradable. In comparison, higher fluorescent intensity of HPI fraction was detected in U-SS supernatant, which represented readily-biodegradable materials. This result also proved the effectiveness of ultrasonic pretreatment and explained the higher organic matter removal rate in U-SS MEC.

Regarding the sludge EBOM, the influent EBOM of WAS MEC was rich in aromatic proteins in various fractions. Whereas HPI, HPO-A, and TPI-A fractions were in the majority of the influent EBOM of U-SS MEC. In the effluent EBOM, fluorescent intensity decreased significantly, revealing the degradation of unsaturated and aromatic organic compounds in MEC. Due to the low organics removal rate, the effluent EBOM from WAS MEC showed a relatively high fluorescent intensity.

### Microbial Community Analysis

A total of 239,472 sequences were analyzed over three samples (Table 3). OTUs at 3% distance were the most detected ones in the biofilm from WAS MEC (5351), with the highest diversity (Shannon index of 5.5624), whereas being the least detected in the raw sludge (WAS, 4786), showing a reduced diversity (Shannon index of 5.436) (Table 3). Similar results were observed for ACE and Chao1 indices (Table 3). Noteworthy, an increase of diversity was detected in anode biofilm communities, indicating an interactive effect between communities during microbial electrolysis.

**Table 3:** Detected number of sequences, OTUs and diversity.

	WAS	WAS-MEC	U-SS MEC
Seq No.	69120	78605	91747
OTUs	4786	5351	4917
ACE index	29544.2	33048.3	31852.3
Chao 1 index	17563.4	19785.6	18717.6
Shannon index	5.436	5.5624	5.0982
Simpson index	0.0387	0.0226	0.0387
Coverage	0.95	0.96	0.96


**FIGURE 4:**
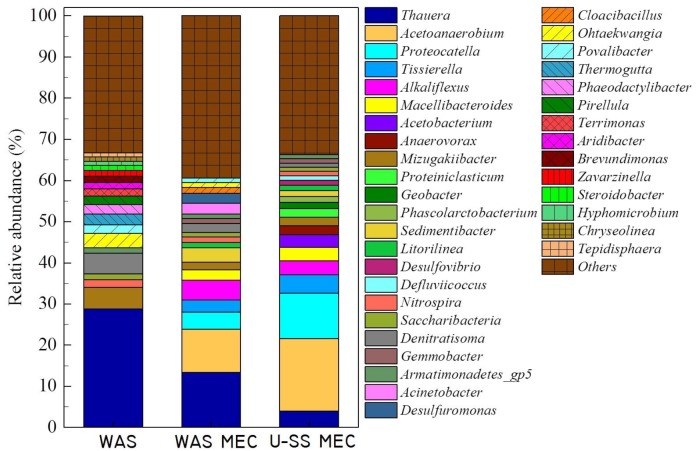
Microbial community structure on the anode of MECs treating WAS and U-SS at genus level.

Characterization of the anode biofilm from the MEC fed WAS (Figure 4) showed it was dominated by *Thauera* (accounted for 10.4% of the population), *Acetoanaerobium* (accounted for 8.9% of the population), *Alkaliflexus* (accounted for 4.1% of the population), *Proteocatella* (accounted for 3.4% of the population), *Sedimentibacter* (accounted for 3.0% of the population), *Tissierella* (accounted for 2.5% of the population), *Acinetobacter* (accounted for 2.2% of the population), *Macellibacteroides* (accounted for 2.0% of the population), *Desulfuromonas* (accounted for 2.0% of the population). The anode biofilm sample from U-SS MEC comprised principally of *Acetoanaerobium* (15.4%), *Proteocatella* (9.6%), *Tissierella* (3.9%), *Thauera* (3.4%), *Alkaliflexus* (3.0%), *Macellibacteroides* (2.9%), *Acetobacterium* (2.6%), *Anaerovorax* (1.9%). The results showed a different composition of populations for the anode biofilm in MECs compared with raw WAS, i.e., an enrichment of *Desulfuromonas, Proteocatella, Alkaliflexus*, and *Acetoanaerobium* along with decrement of *Thauera*. The *Shewanella* and *Geobacter* could convert organic matters to electricity (Lovley, 2006). Low numbers of clones belonged to *Geobacter* was detected and *Shewanella* was not found in raw WAS. However, a high relative abundance (1.3%) was gained for *Geobacter* in the U-SS MEC, which indicated that *Geobacter* was one of the dominant exoelectrogens.

The major part of raw WAS was found to be aerobic bacterial. For example, *Litorilinea* and *Terrimonas* were reported to have capable of oxidizing hydrocarbons (Kale et al., 2013). Similarly, it was reported that *Nitrospira* has capacity of nitrification (van Kessel et al., 2015). A small portion of anaerobic and anaerobic bacterial was also detected, among which *Thauera* and *Mizugakiibacter* were known to proceed denitrification (Mechichi et al., 2002; Watanabe et al., 2015). In contrast, the majority of anode biofilm in MEC was found to be anaerobic and anaerobic bacterial. For example, *Proteocatella* and *Alkaliflexus* were capable of fermentation to produce VFAs (Zhilina et al., 2004; Pikuta et al., 2009); *Nitrospira* had capacity of nitrification (van Kessel et al., 2015); *Thauera* and *Mizugakiibacter* were capable of denitrification (Mechichi et al., 2002; Watanabe et al., 2015); *Desulfovibrio* was recognized as sulfate-reducing bacterium (Gilmour et al., 2011); *Desulfuromonas* and *Geobacter* were responsible for the dissimilatory metal reduction (Roden and Lovley, 1993; Sun D. et al., 2014). Moreover, the growth of *Geobacter* was expected, since acetate presented in sludge SCOD could be adopted as carbon source to grow the exoelectrogenic biofilm on the anode (Yates et al., 2012). The diversity of microorganisms in MEC participated in the degradation of complex organic matters presented in the sludge, as revealed by an important portion of unclassified genus obtained from the anode biofilm sample. More than 11 kinds of aerobic bacterial was also detected on the anode biofilm in WAS-MEC with very low relative abundance (<1%), except for the *Acinetobacter* with a relative abundance of 2.2%, one specie of which was found to be electrochemically active (Xiao et al., 2013). Nevertheless, fewer types of aerobic bacterial (approximately 7 types) were presented in the biofilm of U-SS MEC, indicating the population dominated by anaerobic types. This was in accordance with Borole et al.’s (2011) statement that exoelectrogens in the BES (bio-electrochemical system) were mostly comprised of anaerobic microorganisms, although some bacterial, such as *Shewanella*, was able to grow in the anoxic condition. The better performance of U-SS MEC may be ascribed to the high relative abundance of exoelectrogens.

## Practical Implications of This Work

Syntrophy is recognized as an essential intermediary step in the anaerobic metabolism, especially for the complete decomposition of complex polymers such as polysaccharides, proteins, nucleic acids, and lipids (Liu Q. et al., 2016). The pyrosequencing analysis revealed the coexistence of fermentative and exoelectrogenic bacteria in the anodes, including proteolytic and saccharolytic bacteria, hydrogen-producing bacteria, and so on. This result was in accordance with the finding by Montpart et al. (2015), which discovered the coexistence of different kinds of bacteria irrespective of the substrate fed into the MEC. Other trophic groups in MECs belonged to acetogenic bacteria, hydrogenotrophic methanogenic archaea (Kouzuma et al., 2015; Liu Q. et al., 2016). It has been found that interaction between microbes can improve system performance and energy recovery efficiency (Liu W.Z. et al., 2016). Future works on the level and mechanism of interactions between these syntrophic consortia needs to be disclosed.

The pyrosequencing results also demonstrated that community composition reassembled with inconsiderable structure changes between WAS-MEC and U-SS MEC, despite appearance of *Bellilinea* and *Longilinea* in the U-SS MEC, which may be caused by microbial competition. These results suggest that electron generation and hydrogen production was enhanced by enrichment of cytochrome genes and exoelectrogens involved, rather than community structure adjustments, which arose from the ultrasound pretreatment. Based on this study, the efficiency of a pretreatment method for MEC could be judged from three aspects. The first aspect was the production rate of desirable compounds for the subsequent microbial electrolysis. An effective pretreatment should accelerate the hydrolysis and conversion of organic matter (Beegle and Borole, 2017), for example, VFAs, which were favored for the hydrogen production (Guo et al., 2013; Kadier et al., 2014; Liu Q. et al., 2016). The second aspect was the content of treated sludge, the overall performance of WAS cascade utilization in MEC was substantially related to the microbial community structures, which in turn depended on the content of treated sludge. The U-SS herein resulted in the dominance of *Acetoanaerobium* and *Proteocatella*, whilst alkaline pretreated sludge led to the dominance of *Proteiniclasticum* and *Parabacteroides* in MECs (Liu W.Z. et al., 2016). In addition, the content of treated sludge would supply higher conductivities to the solution, which facilitated mass transfer in anode biofilm. The third aspect was for the economic consideration. Efforts should be tried to balance the energy recovered from biomass and the input energy, as a high conversion rate of COD into energy and a low energy consumption were favorable. The future prospect of this technology relied greatly on this condition, as anaerobic biomethod is important to realize the potential of energy-sufficient in waste/wastewater treatment.

## Conclusion

This study demonstrated bioelectrochemical enhancement of biogas production and organics degradation for WAS by ultrasonic pretreatment in single-chamber MEC. At applied voltage of 0.5 V, biogas production was enhanced 3.68-fold, and cathodic hydrogen recovery was enhanced 2.56-fold, in the MEC fed with ultrasound-pretreated sludge. Meanwhile, the removals of SS and VSS were enhanced by 1.38-fold and 1.48-fold, respectively. Regarding sludge organics, HPI was the main biodegradable substance in MEC, which was improved by the pretreatment. The 16S rRNA pyrosequencing technology was successfully applied to analyze the microbial community, and demonstrated multiple syntrophic interactions between fermentative bacteria, exoelectrogenes, and methanogenic archaea in MEC cascade utilization of WAS and U-SS. The pretreatment facilitated the transformation of SCOD, rather than community structure adjustments during microbial electrolysis.

## Author Contributions

KH and S-qJ designed and performed the experiments. KH, WC, WW, and FH wrote the manuscript. All authors read and commented on the draft manuscript and agreed to the revised version.

## Conflict of Interest Statement

The authors declare that the research was conducted in the absence of any commercial or financial relationships that could be construed as a potential conflict of interest.

## Abbreviations

η_E_: electricity efficiency
ACE: abundance-based coverage estimator
AD: anaerobic digestion
*C*_E_: coulombic efficiency
DNA: deoxyribonucleic acid
DOC: dissolved organic carbon
DOM: dissolved organic matter
EBOM: extracellular biological organic matter
EEM: excitation-emission matrix
EPS: extracellular polymeric substances
HPI: hydrophilic fraction
HPO-A: hydrophobic acid
HPO-N: hydrophobic neutral
IS: intracellular substance
I_V_: volumetrical current density
MEC: microbial electrolysis cell
MFC: microbial fuel cell
NH_3_-N: ammonia nitrogen
OTUs: operational taxonomic units
PCR: polymerase chain reaction
Q: gas production rate
*r*_cat_: cathodic hydrogen recovery
rRNA: ribosomal ribonucleic acid
SCOD: soluble chemical oxygen demand
SS: suspended solid
TCOD: total chemical oxygen demand
TPI-A: transphilic acid
TPI-N: transphilic neutral
U-SS: ultrasound-treated WAS
VFAs: volatile fatty acids
VSS: volatile suspended solid
WAS: waste activated sludge
